# Interplay between Dioxin-Mediated Signaling and Circadian Clock: A Possible Determinant in Metabolic Homeostasis

**DOI:** 10.3390/ijms150711700

**Published:** 2014-07-01

**Authors:** Chun Wang, Zhi-Ming Zhang, Can-Xin Xu, Shelley A. Tischkau

**Affiliations:** 1 Department of Pharmacology, Southern Illinois University School of Medicine, Springfield, IL 62702, USA; E-Mails: cwang@siumed.edu (C.W.); cxu@siumed.edu (C.-X.X.); 2 Department of Anesthesiology, Institute of Translation Medicine, the First People’s Hospital of Chenzhou, Chenzhou 423000, China; E-Mail: otc0735@gmail.com

**Keywords:** dioxin, aryl hydrocarbon receptor, circadian clock

## Abstract

The rotation of the earth on its axis creates the environment of a 24 h solar day, which organisms on earth have used to their evolutionary advantage by integrating this timing information into their genetic make-up in the form of a circadian clock. This intrinsic molecular clock is pivotal for maintenance of synchronized homeostasis between the individual organism and the external environment to allow coordinated rhythmic physiological and behavioral function. Aryl hydrocarbon receptor (AhR) is a master regulator of dioxin-mediated toxic effects, and is, therefore, critical in maintaining adaptive responses through regulating the expression of phase I/II drug metabolism enzymes. AhR expression is robustly rhythmic, and physiological cross-talk between AhR signaling and circadian rhythms has been established. Increasing evidence raises a compelling argument that disruption of endogenous circadian rhythms contributes to the development of disease, including sleep disorders, metabolic disorders and cancers. Similarly, exposure to environmental pollutants through air, water and food, is increasingly cited as contributory to these same problems. Thus, a better understanding of interactions between AhR signaling and the circadian clock regulatory network can provide critical new insights into environmentally regulated disease processes. This review highlights recent advances in the understanding of the reciprocal interactions between dioxin-mediated AhR signaling and the circadian clock including how these pathways relate to health and disease, with emphasis on the control of metabolic function.

## 1. Introduction

Biological life is a complicated and extraordinary process that co-exists in a coordinated, rhythmic symphony with the external environment. Perhaps the most consistent environmental influence on life is the rotation of the Earth about its axis, partitioning each 24 h day into a recurrent period of light and darkness. The environmental pressure of the solar day is so influential that organisms have incorporated this timing feature into their genomes, in the form of a molecular clock that allows synchronization of physiological function with the diurnal rhythms of the environmental light cycle. When this biological clock is constitutively disrupted or the expression and rhythm of genes controlling the circadian clock are disturbed by certain factors, like shift-work, organisms cannot rectify variations in external conditions with internal clock status, leading to pathologic states, including metabolic syndrome, immune-related and neoplastic disease and others [1,2,3,4]. It is increasingly recognized that maintaining the health of the intrinsic molecular clock, which controls the periodic secretion of hormones and enzyme activities, metabolism and food intake, is critical to human health.

The rising prevalence of industrialization and its associated escalation in anthropogenic environmental chemical contamination is consequent with a global increase in disorders of endocrine and metabolism-related diseases, breast and prostate cancer, obesity and diabetes [5,6,7]. Among the numerous chemical contaminants, dioxins and other dioxin-like compounds are unintentional by-products of industrial processes and found ubiquitously in the environment, including in processed food. Thus, humans are exposed to dioxins and dioxin-like chemicals on a daily basis. The toxic effects of dioxins and dioxin-like compounds are predominantly mediated through activation of a ligand-activated transcription factor, aryl hydrocarbon receptor (AhR). After activation by xenobiotic agonists such as the prototypical dioxin, 2,3,7,8-tetrachlorodibenzodioxin (TCDD), AhR can form a heterodimer with its partner, aryl hydrocarbon receptor nuclear translocator (ARNT), to act as a transcription factor complex by binding to xenobiotic/dioxin response elements (XRE/DRE) in the promoters of target genes. Drug-metabolizing enzymes, such as cytochrome P450 1A1 and 1B1 (CYP1A1 and CYP1B1) are common targets of the AhR/ARNT complex. From a toxicological perspective, activation of AhR by a xenobiotic leads to activation of these metabolic enzymes, whose primary function is to feed back and degrade the AhR ligands. Thus, the system works to limit the activity of the offending xenobiotic chemical.

Interestingly, AhR and ARNT are highly homologous to the core circadian clock genes, *Circadian Locomotor Output Cycles Kaput* (*CLOCK*) and *Brain Muscle ARNT-like protein 1* (*BMAL1*). AhR, ARNT, CLOCK, and BMAL1 are all members of the basic helix–loop–helix (bHLH)/period (PER)–ARNT–single-minded (SIM) (PAS) domain-containing family of proteins. PAS-domain containing proteins are critical in development and in adaptation to the environment, including their prominence as regulators of circadian rhythms, as mediators of responsiveness to hypoxia and in xenobiotic metabolism [8,9]. The PAS domain is a protein-protein interaction domain that allows physical interaction among members of the family; PAS domain interactions form heterodimeric transcription factors that act to alter the transcription of target genes. PAS domains are somewhat promiscuous, allowing for multiple different functional partnerships among various family members. The homology of structure and promiscuity in interaction led to the hypothesis that AhR and ARNT might functionally interact with members of the circadian clock gene family. Subsequent studies revealed that AhR not only mediates xenobiotic metabolism by regulating phase I/II metabolizing enzymes, but also controls chemical compound -disrupted circadian rhythm [10,11,12]. Disruption of the circadian clock network also changes host responsive to environmental stimulators, including dioxin-mediated AhR signaling [13,14], thereby indicating that there is a reciprocal relationship between the AhR signaling pathway and the circadian clock. Because both clock disruption and AhR activation are linked to similar disease processes, it becomes compelling to determine whether these integral cellular processes can interact in healthy and diseased cells. New information regarding the interplay between dioxin-mediated AhR signaling and the circadian clock will add to our understanding of environmental toxicant-mediated pathophysiology of metabolic and immune-related diseases.

## 2. Circadian Rhythms in Physiology

**Figure 1:**
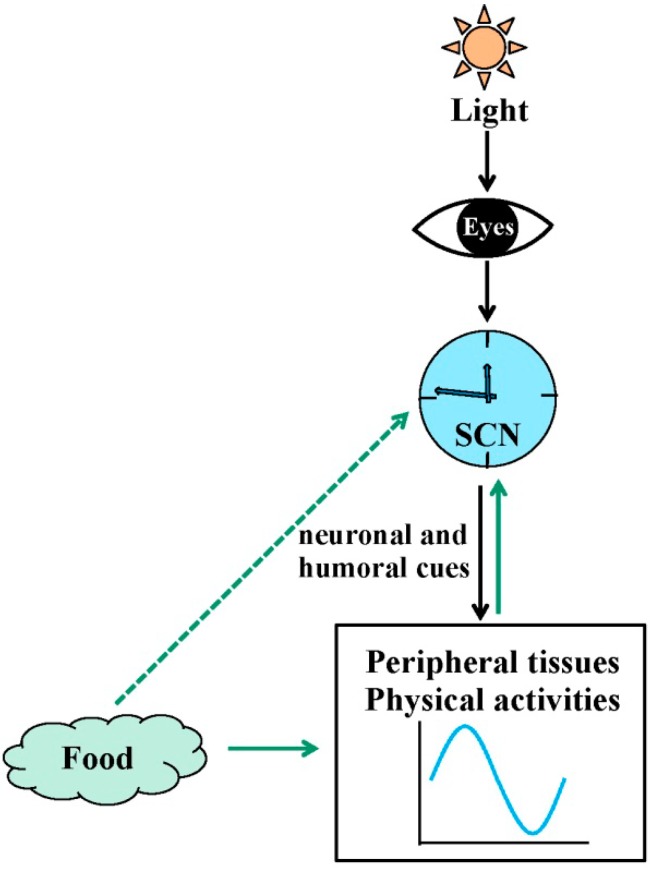
Resetting signals for the central and peripheral clocks. The suprachiasmatic nuclei (SCN) synchronize peripheral oscillators and physiological activities including metabolism, locomotor activity, the sleep-wake cycle, blood pressure, and body temperature via neuronal and humoral cues. Light, food, and feeding regimens affect the central clock in the SCN and/or peripheral clocks.

A circadian clock that exists in nearly all organisms is a critical regulator of myriad physiological and behavioral processes. In mammals, the circadian clock comprises a complex network with a principle oscillator located in the suprachiasmatic nucleus (SCN) of the hypothalamus, and peripheral clocks in nearly every cell, tissue and organ system [15] (Figure 1). The central clock in the SCN, also called the master pacemaker, is an endogenous, autonomous oscillator that functions to maintain physiological synchrony of biological processes with the external environment. The strongest source for biological entrainment to the environment is the light/dark cycle. Light input to the retina impinges upon the optic nerve, which provides direct, monosynaptic input to the SCN through the retinal hypothalamic tract, where the neuronal signal is encoded primarily in the form of glutamatergic neurotransmission. Glutamate signaling sets the phase of clock gene expression by acting upon subset of light-responsive SCN neurons. The SCN, therefore, receives information regarding environmental illumination, processes that signal and then conveys timing information to the rest of the brain and body. The SCN transmits circadian information to control various rhythmic physiological functions such as locomotor activity, the sleep-wake cycle, blood pressure, and body temperature. SCN timing information synchronizes the phases of peripheral clocks via a variety of outputs, through neuronal and humoral cues [16]. In addition to light signals, other physiological factors, such as feeding cues, also entrain the circadian clock through energy metabolism and hormone secretion [17,18,19,20,21]. However, peripheral clocks can still function in rat liver suspension culture and in the SCN lesioned mouse [22,23], indicating that the peripheral clocks possess autonomous molecular clock oscillators, and under normal physiological conditions, the prominent role of the SCN is to drive and synchronize the rhythms among most other tissues. The exact nature of the relationship between the central and peripheral clocks is still being investigated, but the synchronization between SCN-mediated central clock and each peripheral organ-mediated peripheral clock guarantees coherent and harmonious rhythms in the organism.

**Figure 2:**
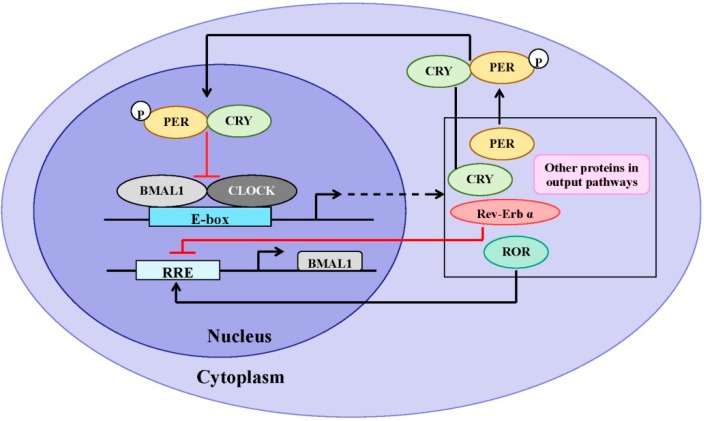
Transcriptional/translational feedback loops (TTFL) model of the molecular clock in mammals. The core clock genes, Brain Muscle ARNT-like protein 1 (BMAL1) and Circadian Locomotor Output Cycles Kaput (CLOCK), heterodimerize and bind to the E-box element on circadian target genes to activate transcription, including period (PER) (homologs: 1–3), cryptochrome (CRY) (homologs: 1 and 2), retinoid-related orphan receptor (ROR), Rev-Erbα and other genes in output pathways, constructing the positive arm of transcriptional/translational feedback loops (TTFL). Phospho-PER and CRY form a complex and inhibit BMAL1/CLOCK-driven transcription, constructing the negative feedback loop. ROR and Rev-Erbα also modulate the activity of the loops through increasing and inhibiting *BMAL1* gene expression, respectively.

At the molecular level, the mammalian circadian clock exists as interlocked, autoregulatory transcriptional/translational feedback loops (TTFL) [15,24,25], consisting of a core group of clock and clock-controlled genes (Figure 2). The central circadian proteins CLOCK and BMAL1 heterodimerize and drive transcription through interactions with E-box *cis*-regulatory enhancer sequences located in the promoter of target circadian genes including Period (PER) (homologs: 1, 2, and 3) and Cryptochrome (CRY) (homologs: 1 and 2). Thus, CLOCK and BMAL1 are considered the positive arm of the TTFL. PER and CRY proteins accumulate; PER acquires progressive phosphorylation under the influence of casein kinase-1δ/ε and subsequently forms a complex with CRY. Then, the PER/CRY complex is translocated into the nucleus where it acts to attenuate the activity of CLOCK and BMAL1, inhibiting CLOCK/BMAL1-mediated transcription, thereby completing a negative feedback loop. The cycle takes approximately 24 h to complete, thus providing the circadian period. The nuclear orphan receptors retinoid-related orphan receptor (ROR) and Rev-Erbα are also transcriptionally regulated by the CLOCK/BMAL1 heterodimer, and modulate the activity of the primary loop through stimulating and inhibiting BMAL1 gene expression, respectively [26], which is considered an accessory loop in the molecular clock system. In addition, the CLOCK/BMAL1 heterodimer also directly regulates the transcription of other genes containing E-box elements in their promoter region, including albumin d-site-binding protein (DBP) [27], vasopressin [28], prokineticin 2 [29] and PPARα [30,31], thereby imposing a circadian regulation upon these genes leading to diurnal rhythmicity in a number of physiological and behavioral events. Circadian clock-controlled output pathways also include many enzymes and regulators involved in metabolism and in xenobiotic detoxification [32].

## 3. Similarities of Structure and Expression Pattern of Dioxin Receptor and Clock Genes

The PAS domain is a prominent feature in proteins that mediate adaptive responses to the environment, including environmental chemicals (AhR), hypoxia (HIF1α) and circadian rhythms (PER, CLOCK and BMAL1) [33,34]. PAS is a structural motif that mediates protein-protein interactions and is named for the three founding members, PER, ARNT and SIM [8]. PER is a core circadian clock regulator [35]; ARNT is required for AhR-mediated responsiveness to environment pollutants, such as dioxins [36]; SIM is a master regulator of midline development in flies [37]. Several PAS domain-containing proteins also contain bHLH motifs located immediately adjacent to their PAS domain on the *N*-terminal side. The bHLH/PAS domain-containing proteins can form homodimers or heterodimers with other bHLH/PAS proteins. Dimerization is typically followed by binding to specific regulatory elements in target genes; thus bHLH-PAS dimers function as transcription factors. Amino acid sequence analysis of the AhR protein reveals that amino acids 28 to 83 encode the bHLH motif, and the regions from amino acids 119 to 17 and from amino acids 282 to 335 contain PAS domains [38]. Furthermore, BMAL1 shows the highest similarity to ARNT and ARNT2 (44.3% and 41.6%, respectively) in the bHLH and PAS domains among PAS proteins [39]. These observations prompted the hypothesis that functional crosstalk may occur between AhR/ARNT and the molecular circadian clock. Subsequent studies demonstrated that, in addition to forming a functional transcription factor by binding ARNT, AhR can also bind with BMAL1. The data suggest that AhR/BMAL1 heterodimers can compete with CLOCK/BMAL1 heterodimers for binding to the E-box in the promoter of the *PER1* gene in mouse hepatoma Hep1c1c7 cells [40] although two-hybrid experiments show that BMAL1 fails to form a complex with AhR [41]. Unlike CLOCK/BMAL1, which activates the Per1 promoter, AhR/BMAL1 will inhibit activity at the E-box, thereby suppressing Per1 transcription.

AhR is widely expressed in embryonic and adult mice including in the suprachiasmatic nucleus (SCN) [42,43]. Similar to circadian clock genes containing bHLH/PAS domains, AhR and ARNT protein levels display diurnal changes in liver, lung and thymus tissues of female Sprague-Dawly rats [44]. Furthermore, the daily cycles of AhR and ARNT protein exhibit an identical oscillation pattern in liver, *i.e.*, they both have dual peaks at zeitgeber time 5 (ZT5, 5 h after lights on) and ZT22. AhR protein expression patterns in liver, lung and thymus are remarkably similar. AhR mRNA in liver, pituitary and SCN of rats and mice has a prominent diurnal rhythm with a single peak [32,45,46]. The mechanisms for the difference of AhR protein and mRNA expression pattern are unknown. In addition, the mRNA levels of AhR-induced drug-metabolizing enzymes also show rhythmic expression in liver, pituitary and SCN [32,45,46], suggesting that AhR signaling may be under the physiological control of the circadian clock. In addition, changes in clock gene expression and circadian clock function observed after activation of AhR implicate AhR signaling as a regulator of the circadian clock. The importance of the reciprocal interactions of these pathways in health and disease remains relatively unknown and comprises an active area of exploration.

## 4. Effects of Circadian Clock on AhR Signaling

**Figure 3:**
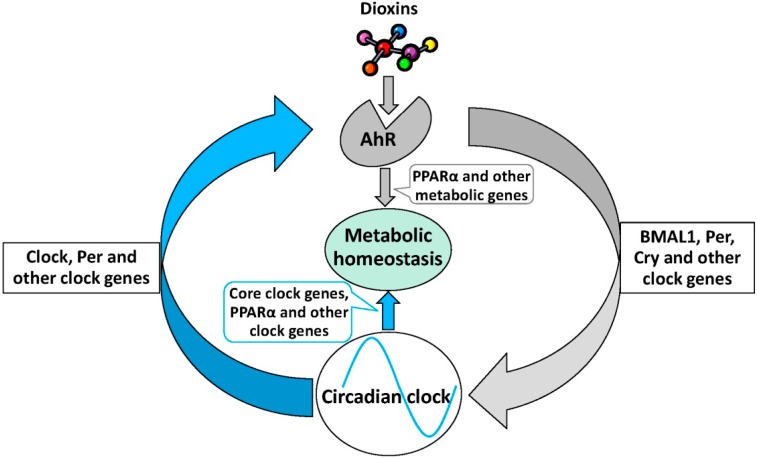
A simplified model depicting the crosstalk between dioxin-mediated AhR signaling and the circadian clock in metabolic homeostasis. Dioxin exposure and circadian clock disruption can both impair metabolic homeostasis through regulating the expression of core clock genes and metabolic genes displaying circadian rhythmicity. Dioxin-mediated AhR signaling disrupts clock function in the SCN and in peripheral tissues, but AhR is reciprocally regulated by the circadian clock.

Although the precise mechanisms by which the molecular clock regulates the diurnal expression of AhR signaling proteins remain unclear, disruption of clock genes, *PER1* or *PER1*/*PER2*, increases 2,3,7,8-tetrachlorodibenzo-*p*-dioxin (TCDD)-induced CYP1A1 and CYP1B1 expression in mammary gland and liver of mice [13,47]. Furthermore, the *PER1*, *PER2* and *PER1*/*PER2* mutations abolish the diurnal variation of TCDD-induced CYP1A1 expression in mammary gland and liver of mice [14]. However, inhibition of PER2 alone, using an siRNA approach, significantly decreases both induction of the P450 enzymes as well as AhR and ARNT expression in TCDD-treated Hepa1c1c7 cells [47]. These results suggest that AhR regulation by circadian clock is complex. More importantly, another group has reported that *CLOCK* mutant mice show decreased expression of AhR mRNA and an inhibition of benzo [α] pyrene-induced CYP1A1 expression [48]. The presence of an E-box element, a binding site for CLOCK/BMAL1, in the AhR promoter [49] suggests that the CLOCK/BMAL1 heterodimer may directly regulate AhR transcription. These data provide clear evidence that the circadian clock system may be involved in regulation of AhR/ARNT rhythmicity and dioxin-mediated AhR signaling although the exact mechanism(s) has yet to be determined. Furthermore, these studies suggest that the primary transcriptional loop of the circadian clockworks, of which CLOCK and PER are critical elements, may play a role in determining the biological outcome of xenobiotic exposure (Figure 3).

## 5. Effects of AhR Signaling on Circadian Rhythm

The emerging picture resulting from investigation of the effects of AhR signaling on the function of the endogenous circadian clock suggests a relationship between AhR and the molecular clock that is context-specific and tissue-dependent. The period of the endogenous circadian rhythm of behavioral activity is fairly normal in AhR^−/−^ mice, as is the ability of these animals to entrain to a typical light/dark cycle. Although AhR deficiency may have little effect on innate behavioral circadian rhythms in the absence of exogenous agonists, exposure to the high affinity AhR ligands, TCDD or β-naphthoflavone (BNF), decreases the behavioral response of the animals to light signals that can reset the central clock and alter expression of Per1 and Bmal1 in both SCN and liver [46,50]. Furthermore, the high affinity AhR ligand derived as a photoproduct of tryptophan (Trp) metabolism, 6-formylindolo [*3*, *2-b*] carbazole (FICZ) alters the circadian expression of clock genes (*PER1*, *CRY1*, and *CRY2*) in SCN 2.2 cells, and inhibits glutamate-induced phase shifting of the mouse SCN electrical activity rhythm *in vitro*, which is a mean to explore the neurochemical effects of light on the SCN [51]. Collectively, these data indicate that AhR activation suppresses the responsiveness of the circadian clock to light. This may indicate that dioxin exposure can inhibit the plasticity of the clockworks, making the exposed organism less capable of responding to critical environmental stimuli. AhR activation inhibits basal or light-induced PER1 expression in liver and SCN of mice through interaction with BMAL1 [40] and activation of JNK [50], respectively, providing a hint at the molecular mechanism by which AhR signaling regulates the circadian clock. Similarly, TCDD alters expression of gonadotropin releasing hormone (GnRH) in the rat hypothalamic GnV-3 cell line through deregulation of Per1 [52]. These data suggest that PER1 is a critical molecular link between dioxin-mediated AhR signaling and the circadian clock system. TCDD exposure also alters the expression of other genes involved in the circadian rhythm [53] and the rhythm of circadian clock genes in the mouse ovary [54] and murine hematopoietic stem cells [55]. In addition to TCDD, other environmental chemicals also affect circadian function. For example, Dibenz [*a*,*h*] anhracene (DB [*a*,*h*] A) affects the expression of genes involved in circadian rhythm [56]. Conversely, one study shows that TCDD exposure does not affect the circadian system through measuring bioluminescence from explanted tissues of PER2::Luciferase mice [57]. It is possible that TCDD does not affect PER2 expression, and therefore, the PER2::Luciferase mice provide a poor model for examination of interaction of AhR with the circadian clock. Collectively, the aggregate data provide evidence that environmental contaminants may upset the physiologic balance of the circadian clock system. Thus, cross-talk between AhR and the molecular circadian clock may be important in regulating homeostatic function of peripheral and central clocks (Figure 3).

## 6. The Role of AhR Signaling in Metabolic Homeostasis

The circadian clock is critical in maintaining metabolic homeostasis [1,2]. Similarly, AhR activation is associated with metabolic imbalance, leading to development of metabolic syndrome. Our recent studies have demonstrated that AhR knockout blunts the expression rhythm and decreases the basal expression of peroxisome proliferator-activated receptor-α (PPARα) and a series of other metabolically important genes in the mouse liver. Furthermore, AhR knockout mice display an altered blood glucose rhythm and enhanced insulin sensitivity [58]. *PPARα*, an important metabolism gene, is robustly rhythmic in the mouse liver [30,32,58], and parallels the rhythmic pattern of AhR. Increasing evidence indicates that PPARα may represent a critical link between the circadian clock and metabolism. As a vital organ for metabolic function including detoxification, digestion, and protein synthesis hepatic circadian rhythmicity may be critical for overall peripheral circadian physiology. Our data suggest that a physiological function of the AhR may include regulation of circadian metabolism through peripheral clock genes, specifically those that lie at the interface of the circadian system and metabolism. Furthermore, activation of AhR by TCDD alters rhythms in activity, feeding [59,60] and serum hormone secretion [61,62,63] in mice and rats. In addition, benzo [a] pyrene (BaP) exposure alters the circadian rhythm of blood pressure [64]; ultraviolet radiation exposure leads to dysregulation of rhythmicity and homeostasis through AhR-mediated entrainment of circadian clocks [65,66]. Taken together, these data imply that AhR signaling potentially orchestrates the metabolic homeostasis through a crosstalk with central and/or peripheral clock (Figure 3). More investigation is required to explore mechanisms that allow chemical-mediated or endogenous AhR signaling to regulate the clock and its downstream metabolic function.

## 7. Conclusions

Dioxins are a class of typical AhR agonists. Long-term follow-up studies of dioxin exposure have revealed that dioxin exposure contributes to the development of cancers, metabolic disease, endocrine disorders, immune disease and other pathologies [10,67,68,69,70]. Circadian clock disruption can also contribute to metabolic disease, tumor promotion, and other related diseases [1,2,3,4]. Although the precise mechanisms behind metabolic and tumor-generating effects of dioxins have not been elucidated, AhR-mediated circadian clock disruption by AhR ligands in central and peripheral tissue, as suggested by the current review, may be important (Figure 3). In addition, AhR activation can induce Phase I and Phase II metabolizing enzymes. Interestingly, these same metabolic enzymes are also regulated by circadian clock [66,71,72]. In general, the emerging evidence supports significant cross talk between AhR signaling and the circadian clock. The interactions are reciprocal and complex in nature. AhR activation can influence clock gene expression, particularly *Per1*, and the molecular clock can influence expression of several components of the canonical AhR signaling pathway, including both AhR and ARNT. Clearly, a better understanding of these complex mechanisms is necessary for a more complete understanding of the clock and AhR in health and disease. Understanding the relationship between AhR signaling and circadian clock may provide a new insight into mechanisms underlying the development of a variety of disease states, including those associated with energy metabolism and cancer.
